# MD2 AMP(K)s up the link between lipid accumulation and inflammation in NAFLD

**DOI:** 10.1002/ctm2.832

**Published:** 2022-04-22

**Authors:** Anneleen Heldens, Sander Lefere

**Affiliations:** ^1^ Hepatology Research Unit, Department of Internal Medicine and Pediatrics, Liver Research Center Ghent Ghent University Ghent Belgium; ^2^ Gut‐Liver Immunopharmacology Unit, Department of Basic and Applied Medical Sciences, Liver Research Center Ghent Ghent University Ghent Belgium

1

Nonalcoholic fatty liver disease (NAFLD) has become the most common liver disease worldwide, with a global prevalence of 25%.^1^ The disease covers a wide spectrum of histological manifestations, ranging from simple steatosis to nonalcoholic steatohepatitis (NASH) with or without fibrosis. Furthermore, NASH is an important risk factor for the development of hepatocellular carcinoma (HCC). Despite the high prevalence and the impact on public health, no registered drugs for the treatment of NAFLD are available. At the moment, caloric restriction, exercise and bariatric surgery are the most effective treatment options. Therefore, the identification of new therapeutic targets is a major unmet need.^2^

The progression to NASH and liver fibrosis is critical, as this predisposes to HCC and is associated with an increased risk of all‐cause mortality. Activation of Toll‐like receptor 4 (TLR4) plays a crucial role in this progression. TLR4, coupled with myeloid differentiation factor 2 (MD2), is a pattern recognition receptor expressed in the liver by hepatocytes, Kupffer cells and hepatic stellate cells that binds several ligands, including lipopolysaccharide (LPS) and circulating free fatty acids (FFAs). Recognition of LPS or FFA activates the MyD88‐dependent and TIR‐domain‐containing adapter‐inducing IFN‐β (TRIF)‐dependent pathways. MyD88‐mediated signalling results in the activation of nuclear factor‐κB (NF‐κB) and mitogen‐activated protein kinase (MAPK) and the production of proinflammatory cytokines. The TRIF‐dependent pathway activates TANK binding protein‐1 (TBK‐1) and interferon regulatory factor‐3 (IRF‐3).^3^

Previous studies have established a role for TLR4/MD2 in hepatic inflammation, specifically by activating inflammatory pathways in macrophages.^4^ In this issue of *Clinical and Translational Medicine*, Luo et al. identify an additional mechanism by which MD2 contributes to NAFLD progression, specifically lipid accumulation in hepatocytes. First, they showed that MD2 gene expression and protein levels were elevated in both patients and mice with NAFLD. Hepatocytes and myeloid cells were identified as the main cellular source of MD2 in the liver.^5^

The authors confirmed previous findings in preclinical models, showing that MD2KO mice fed a high‐fat diet (HFD) were protected against dyslipidemia and liver steatosis. Next, bone marrow reconstitution studies were performed to distinguish the role of MD2 in both cell types. Both wild‐type (WT) → MD2KO and MD2KO → WT were partially protected against the development of NAFLD, indicating that MD2 in both cell types is important in the development of hepatic steatosis.^5^

On a mechanistic level, the AMP‐activated protein kinase (AMPK) pathway was identified as one of the downstream targets of MD2 in hepatocytes. AMPK is known to suppress sterol regulatory element binding protein‐1 (Srebp1), an important transcription factor regulating fatty acid synthesis. Indeed, in HFD‐fed MD2KO mice, no induction of Srebp1 was observed. In agreement, chromatin immunoprecipitation (ChIP) analysis revealed that MD2KO prevented Srebp1 binding to the promotor regions of its target genes in palmitate‐stimulated primary hepatocytes. By using inhibitors and knockout models, TBK‐1 was found to be the regulating factor in the MD2/AMPK cascade. In summary, hepatocyte MD2 activation causes dephosphorylation of AMPK via TBK‐1 and subsequently drives the expression of Srebp1. Last, Luo et al. uncovered that macrophage MD2‐dependent production of cytokines, tumour necrosis factor‐α (TNFα) in particular, can bypass MD2 on hepatocytes by activating TNF receptor 1 (TNFR1). Downstream, this induces TBK1 phosphorylation and AMPK inhibition, thus constituting a link between hepatic inflammation and lipid accumulation^5^ (Figure 1).

**FIGURE 1:**
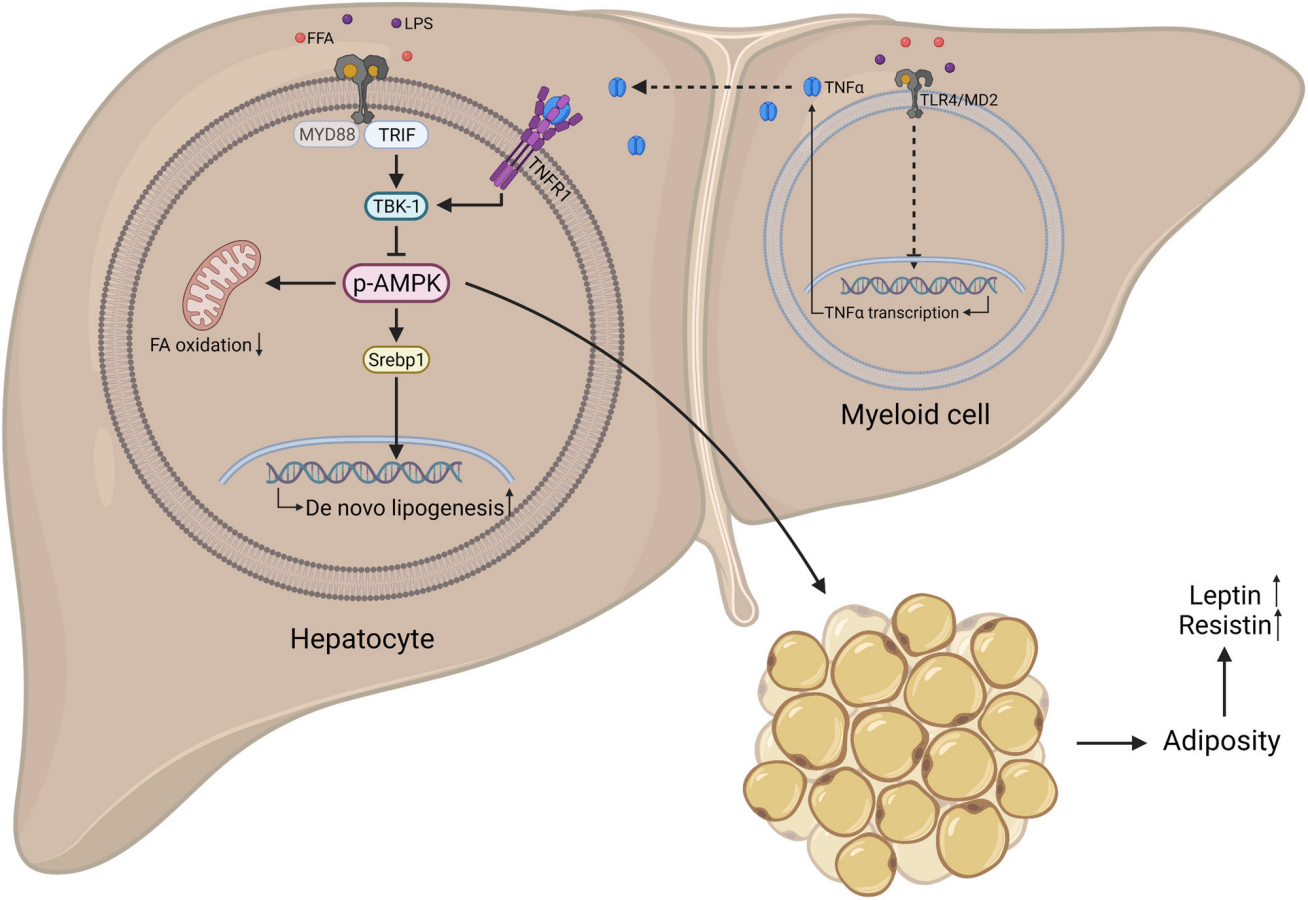
Schematic representation of the TLR4/MD2/AMPK pathway. Stimulation of TLR4/MD2 by FFA and LPS or of TNFR1 by TNFα on hepatocytes inhibits the phosphorylation of AMPK through TBK‐1. AMPK inhibition promotes hepatic steatosis through increased de novo lipogenesis and suppressed fatty acid oxidation. In addition, liver‐specific AMPK can reduce adiposity and decrease levels of leptin and resistin, and thus, AMPK inhibition can further increase obesity, inflammation and NAFLD through a vicious cycle. AMPK, AMP‐activated protein kinase; FFA, free fatty acids; LPS, lipopolysaccharide; MD2, myeloid differentiation factor 2; Srebp1, sterol regulatory element binding protein 1; TBK‐1, TANK binding protein‐1; TNFα, tumor necrosis factor α; TRIF, TIR‐domain‐containing adapter‐inducing IFN‐β; TLR4, toll‐like receptor 4

This compelling study gives rise to several questions in this field. First, apart from hepatocytes and liver macrophages, other cells, including adipose tissue (AT)‐associated macrophages, express these receptors.^7^ As NAFLD is usually seen in patients with obesity, the impact of chronic low‐grade AT inflammation cannot be ignored. AT expansion causes hypoxia and subsequently the release of damage‐associated molecular patterns and proinflammatory cytokines by stressed adipocytes. These inflammatory signals lead to phosphorylation of the insulin receptor and insulin resistance. AT insulin resistance is characterised by inadequate suppression of lipolysis, leading to an increased flux of FFA towards the liver, one of the major causes of hepatic steatosis. Therefore, further studies should examine the contribution of AT and hepatic MD2 using cell‐ and tissue‐specific knockout models.^8^

The second is the central role of AMPK, an energy‐sensing enzyme, activated by an increased AMP/ATP ratio and therefore a promising therapeutic target in NAFLD. In this study, the authors focused on the effect of AMPK on Srebp1, but liver‐specific AMPK activation is known to reduce hepatic steatosis via several pathways, such as downregulation of de novo lipogenesis and stimulation of fatty acid oxidation and autophagy. Moreover, liver‐specific activation of AMPK also produces a variety of extrahepatic effects, specifically resistance to weight gain, reduced adiposity and, as a result, decreased levels of leptin and resistin (Figure 1). These outcomes were either not observed or measured in the current study. In addition, stimulation of AMPK can improve insulin sensitivity, although these effects are limited in time when HFD consumption is prolonged.^6^

A general concern is that as the role of the hepatocyte TLR4/MD2/TRIF/TBK1 pathway in hepatic steatosis is identified, the optimal drug target is still unclear. Unfortunately, a TLR4 antagonist, JKB‐121, did not achieve its primary end point of liver fat content reduction in a phase II trial, while there were also safety concerns.^2^ In addition, several studies have been carried out targeting the TRIF pathway in hepatocytes, but depending on the model, TRIF knockout could protect against or worsen inflammation. In diet‐induced models of NASH, TRIF knockout seems to worsen steatosis, inflammation and fibrosis. Meanwhile, in acute models of hepatocyte cell death, mice were partially protected against the inflammatory response. It has been suggested that TRIF knockout can stimulate the MyD88‐dependent pathway and thus cytokine production in hepatoyctes.^9^ Therefore, investigating downstream targets, such as TBK‐1, as therapeutic targets might be more appropriate, as this could also inhibit TNFα‐mediated hepatocyte lipid accumulation. As discussed, AMPK is another attractive option, although caution is advised, as prolonged activation of AMPK can lead to side effects, including kidney dysfunction and hyperphagia.^6^

Overall, the promising work presented by Luo et al. reveals the double role played by the MD2/AMPK/TBK1 pathway in hepatic lipid accumulation. If confirmed, modulation of the key mediators might emerge as a new therapeutic approach.

## CONFLICTS OF INTEREST

The authors do not report any conflicts of interest.
